# Willingness to Use Electronic Medical Record (EMR) System in Healthcare Facilities of Bahir Dar City, Northwest Ethiopia

**DOI:** 10.1155/2020/3827328

**Published:** 2020-08-26

**Authors:** Birhanu Berihun, Desta Debalkie Atnafu, Getachew Sitotaw

**Affiliations:** Department of Health System and Health Economics, School of Public Health, College of Medicine and Health Sciences, Bahir Dar University, Bahir Dar, Ethiopia

## Abstract

**Background:**

Globally, electronic information and communication technology has been applied and much expanded in the healthcare industry. However, in developing counties including Ethiopia, EMR system adoption and utilization for medical practice are still inconsistent, and healthcare institutions which started utilization currently have also failed to sustain. A desirable readiness of healthcare experts is mandatory to expand digital health service delivery. Thus, this study is aimed at estimating the proportion of the willingness of professionals in Bahir Dar city to use EMR and at identifying factors associated with this proportion.

**Methods:**

An institution-based cross-sectional study was conducted from September 1 to October 30, 2019, among 634 health professionals. Respondents were selected using a simple random sampling method. Data were entered into EpiData version 3.1 and exported to SPSS version 23 for further analysis. Descriptive statistics were computed to describe study variables and presented using tables. Willingness to use the EMR system was computed. Bivariable and multivariable binary logistic regression models were fitted to identify the associated factors. The odds ratio with 95% confidence interval was used to measure the strength of association.

**Results:**

A total of 616 health professionals participated in the study with a response rate of 97%. The proportion of willingness to use the EMR system was 85.9%. Among health professionals who were not willing to use EMR, lack of access to EMR training (73.4%) was a major barrier to the willingness to use EMR. A multivariable logistic regression analysis showed that those health professionals who had good computer skill (AOR = 2.5; 95% CI: 1.3-4.6), good knowledge on EMR (AOR = 2.1; 95% CI: 1-4.4), gotten EMR training (AOR = 3.8; 95% CI: 1.7-8.1), EMR guideline access (AOR = 2.8; 95% CI: 1.4-5.6), and management support (AOR = 2.6; 95% CI: 1.4-4.8) were more likely willing to use the EMR system.

**Conclusions:**

Majority of the professionals were willing to use the EMR system. EMR program should involve computer illiterate, less knowledgeable, those unable to access EMR guidelines, and managerially unsupported professionals. Enhancing health professionals' attitude and contextualizing EMR training in the healthcare curricula are highly recommended to scale up EMR use.

## 1. Background

To date, the application of electronic information and communication technology (ICT) in the healthcare system has increased worldwide. These include telehealth, mobile health applications, electronic medical records, and health information management systems [1]. A survey conducted by the World Health Organization (WHO) in 2012 indicated 45% of countries used electronic systems for patients' data management. Besides, 30% of countries have been collecting and communicating patient information via electronic systems [2, 3]. In Ethiopia, the five-year perspective strategic plan known as health sector transformation plan, which had been implemented from 2015 to 2020, envisioned utilization of electronic health management information system and strengthening the electronic medical record (EMR) system in the healthcare industry.

EMR, which is a patient's health and health-related information record data set system, is operating based on an application of computer software. In other words, it is as an electronic software program developed for the storage, processing, and exchange of medical and medical-related information, and the patients' data can be created, gathered, managed, and consulted by authorized clinicians or staff within healthcare organizations [4, 5].

EMR systems in the medical sectors do have numerous advantages where they simplify service delivery and decision-making process [6]. Research conducted in hospitals in the US revealed that EMR system adoption decreased patient length of stay, patient mortality rate, and hospitalization by 0.11%, 0.18%, and 0.46%, respectively, out of 30 days [7]. Another study in Australia showed that 5% more patient consultations per hour had been seen by medical practitioners in the EMR system compared to the paper-based medical recording system [8]. Similarly, results from India specified that the duration of patient treatment was significantly shorter for the electronic record system [6]. This has paved the way for the increased acceptance and implementation of EMR by healthcare systems in the world including resource-limited countries like Ethiopia, where the EMR system has been launched by the Ministry of Health and included in the strategic plan [2, 3, 9].

The EMR system ensures high-quality documentation and easier to retrieve data system than the paper-based medical record system [8]. According to the research finding, retrieving evidences through EMR system was 40% more complete and 20% faster than paper records [6]. A study in Malawi in 2017 also indicated that 76% of health workers preferred to work in healthcare facilities which had installed the EMR system, justifying that the system is fast and easy to use. Additionally, 77.8% of respondents supported that the electronic healthcare data management system was more accurate, and as a result, the patients were served more quickly [10]. On the contrary, paper-based prescriptions had 18.5% of reading error for the actual medication and also had lack of patient confidentiality and privacy due to unauthorized users who can easily access information [4, 6, 11].

A significant amount of medical errors around the globe are accustomed to weak EMR program and data system functioning and willingness to use EMR by health experts. Findings from North Carolina indicated that one in seven Medicare patients suffered harm and 63% of the harms were attributed to hospital medical care although 44% of the medical care errors were preventable if the installed EMR system was used by healthcare professionals enthusiastically.

EMR systems are alarmingly being utilized and automated in healthcare systems to improve effectiveness, timeliness, efficiency, quality of healthcare, data management, and decision-making. Nevertheless, a practice of EMR system implementations has faced confrontations from users even in the technically equipped healthcare working setups [12]. A survey by the WHO in 2016 indicated financial, technical, and infrastructural barriers were the commonest which is directly related to healthcare professionals' unwillingness to use EMR [13]. Similarly, other literatures found that different factors would also potentially affect the willingness to use EMR systems by healthcare experts. These include system users, facility setups, availability of skilled human capital, information and resource availability, training, computer literacy, English language proficiency, educational status, and knowledge for EMR [6, 14–19]. Literature found around the world indicated that healthcare professionals' resistance and dissatisfaction in using new technology like EMR was a major barrier to escalate the electronic health data system [13, 20, 21].

Given the high burden of disease and the increased number of skilled personnel in Ethiopia, information quality and use remain weak, particularly at primary healthcare facilities. This is because of deprived EMR infrastructure aggravated by the unwillingness to use the already available technology [14]. This makes surveillance systems and health communication among different healthcare organizations and professionals very difficult [22]. A survey conducted between 2008-2009 on health personals' willingness to use EMR discovered that 42.3% of physician had willingness to use patients' electronic health records during clinical practices [23]. Whereas, an another study carried out in Saudi Arabia revealed that 83% of healthcare professional preferably use EMR system than paper-based system [19].

The EMR system or smart care software implementation was started in Ethiopia since 2007/2008 with the help of Tulane University [24], whereas the adoption and utilization rate is still insignificant and gone to back [20, 24]. The healthcare institutions that already started to use the EMR system were unable to sustain the EMR system [20, 24]. Most of the strategies in Ethiopia are emphasized on the material aspect particularly the installations and infrastructuring of EMR systems and have ignored the attitudinal and behavioral factors (willingness) of healthcare practitioners in using the EMR system as supported by a study conducted in Ayder Referral Hospital, Northern Ethiopia, which showed that a significant number of professionals (43.3%) had unfavorable attitude towards EMR system use [25].

Although Ethiopia has been trying to implement the EMR system at a small-scale level since 2007/2008, there have been insignificant numbers of studies conducted on EMR among medical practitioners. This study is aimed at assessing the willingness of healthcare professionals in healthcare facilities of Bahir Dar city, Ethiopia, to use EMR and at identifying the factors that are related to the willingness to use the EMR system.

## 2. Methods

### 2.1. Study Setting and Participants

Bahir Dar city, where the study was conducted, is the capital city of Amhara National Regional Government. It is found 480 kms away from Addis Ababa, the capital city of Ethiopia. In the city, there are 10 public health centers, four public and 3 private hospitals, and 5 clinics; however, only six healthcare facilities have started to use the EMR system. Among them, three hospitals: Adinas, Gamby, and Felege-Hiwot; two public health centers: Bahir Dar and Han health centers; and one clinic: Marie Stopes International clinic, implemented the EMR system. In this study, only four of the healthcare facilities were selected and involved in the study. About 984 health professionals were found in those selected health institutions that include Felege-Hiwot Comprehensive Specialized Hospital, Gamby Teaching Hospital, Bahir Dar Health Center, and Marie Stopes International Clinic.

### 2.2. Study Design and Period

A cross-sectional study was conducted between September 1 and October 30, 2019, in healthcare facilities of Bahir Dar city, North West Ethiopia.

### 2.3. Sample Size Determination and Sampling Procedures

The sample size was calculated based on a single population proportion formula using Epi Info version 3.5 with the following assumptions: 95% confidence interval (*a* = 0.05), 5% margin of error in the estimate of willingness to use the EMR system, and taking the proportion (*p*) of willing to use EMR by healthcare professionals, which was 50%. The final sample size estimated, after taking a 10% nonresponse rate and a design effect of 1.5, was 636. Based on the number of professionals found in each healthcare facility, the proportion to size allocation was made to achieve the desired sample size of healthcare professionals in each selected healthcare facility. Multistage sampling was employed to recruit study participants. In the first stage, four out of six health institutions, which started to use EMR, were selected using a simple random sampling method, while the second stage was the selection of the final respondents from the healthcare expert sampling frame available in the human resource department. During the data collection period, the numbers of allocated sample size in the selected facility were further proportional to the number of experts in each healthcare profession. Then, healthcare professionals that participated in the study were identified from each profession by using a computer-generated, simple random sampling method.

### 2.4. Data Collection Tools, Techniques, Procedures, and Quality Management

A structured questionnaire adapted by reviewing the literature was used to collect data through self-administered interviews. The questionnaire was translated into the local language (Amharic) and back-translated to check the consistency. Sociodemographic, skill, technical, and organizational variables were included in the questionnaire. Appropriate training was given for data collectors (health informaticians) and supervisors on the objective of the study, data collection tools, data collection procedures, respondents' approach, and respondents' right prior to the data collection period. Before the actual data collection, the tool was pretested among 5% of the sample size outside the study area with similar characteristics to the respondents, and necessary corrections were done accordingly. The investigators and supervisors closely checked the data collection procedures on the spot. Any questionnaire with a defect was rejected and counted as a nonresponse.

### 2.5. Data Processing and Analysis

Data were coded and entered into EpiData version 3.1 software and then exported to SPSS version 23 for analysis. Frequencies, proportions, and summary statistics were used to summarize the data. Both bivariable and multivariable logistic regression analyses were carried out to identify the association between the dependent and independent variables. The degree of associations between outcome and exposure variables was described by the adjusted odds ratio with a 95% confidence interval (CI). Willingness to use the EMR system is the preparedness of healthcare professionals to use the EMR system. Willingness to use the EMR system by healthcare experts was graded into “yes” and “no” using composite scores obtained from all the five willing to use EMR questions tested and adopted from Khoja et al. [26]. Five of these questions were scored, and the maximum score obtainable was 5 marks. A score of 3 marks and above out of 5 marks suggested willingness while a score of less than 3 marks suggested unwillingness to use the EMR system.

### 2.6. Ethical Considerations

The study was approved by the institutional ethical review committee of the Bahir Dar University, College of Medicine and Health Sciences. Letters of permission were obtained from Amhara regional public health institute. Verbal consent was obtained from each respondent. Each study participant was informed about the purpose and anticipated benefits of the research project. Privacy and confidentiality were guaranteed throughout the study.

## 3. Results

### 3.1. Sociodemographic Characteristics

A total of 616 health professionals participated in the study with a response rate of 97%. The mean age of respondents was 30.9 ± 5.5 years. The majority of respondents (92.7%) were in the age range of 20-39 years. More than half (57%) were female respondents, and about 437 (70.9%) were married. About 453 (73.5%) of the participants were first-degree holders, and 60.9% of them were with working experience of less than 7 years. The majority of the respondents (328 (53.2%)) were nurses by profession (Table 1).

### 3.2. Technical Factors for Willingness to Use the EMR System

Almost half (45.9%) of health professionals had good knowledge towards EMR system use. This is because of lack of planned training packages in healthcare facilities. However, more than half (58.3%) of the respondents had sufficient skill to use computer systems which in turn able them to use the EMR system. The computer skill was the result of a short-term training taken by each expert sponsoring themselves. Thus, the more professionals were computer literate, the more likely skillful they are in using the EMR system. However, more than half (54.9%) of the respondents did not receive EMR system training because of lack of access and plan for training (73.4%), absence of interest for training (9.2%), lack of time to be self-trained (7.7%), etc. More than two-thirds (65.3%) of health professionals had no English language barrier to use a computer and EMR system. This is because of the fact that most of the experts were bachelor and above by profession. Nearly two-thirds of the respondents used a computer device in the EMR system for the following obvious purposes: writing of reports (11.4%), keeping patient files and profiles (54.7%), and reading (28.9%) (Table 2).

### 3.3. Organizational and Resource-Related Factors

Of the total respondents, 484 (78.4%), 538 (87.3%), and 399 (64.8%) were able to access computers, the EMR guideline, and the Internet for the purpose of running an EMR system, respectively. More than two-thirds (66.6%) of healthcare professionals had been supported by trained IT technical personnel recruited for EMR system maintenance. Nearly two third (64.6%) of respondents got managerial support to use EMR system, however, only 38.8% of the respondents replayed that adequate budget was allocated for EMR system (Table 3).

### 3.4. Willingness to Use the EMR System

The proportion of those who were willing to use the EMR system in healthcare facilities was 85.9% (95% CI: 82.3-89.5). About 71.3% and 83.8% of professionals were willing to avail even a personal computer and undergo a computer training in order to enable EMR usage, respectively (Table 4).

### 3.5. Factors Associated with Willingness to Use the EMR System

After adjustment for possible confounders, some variables remained in the multivariable model: healthcare professionals who had trained for EMR system software were 3.75 times more likely to be willing to use the EMR system than those who had never trained for EMR system software (AOR = 3.75; 95% CI: 1.73, 8.12); study subjects who have gotten EMR guideline around the clinical working area were 2.76-fold more likely to have willingness to use the EMR system compared to those with no EMR guideline (AOR = 2.76; 95% CI: 1.36, 5.60); respondents working in the presence of higher management support were 2.59 times more likely to be willing to use the EMR system than their counterparts (AOR = 2.59; 95% CI: 1.40, 4.77); the odds of willingness to use the EMR system was 2.46 and 2.11 times higher in those with good computer skill (AOR = 2.46; 95% CI: 1.31, 4.61) and good knowledge on the EMR system (AOR = 2.11; 95% CI: 1.02, 4.37), respectively (Table 5).

## 4. Discussion

The study is mainly dedicated to assess the willingness to use an EMR system among healthcare experts operating in healthcare facilities of Bahir Dar city, Northwest Ethiopia. Hence, the study proved that 85.9% of the experts were willing to use the EMR system in their assigned clinic and committed to advance patient data management system even by being willing to avail one's own computer in the working setup (71.3%) and taking computer training sponsoring themselves (83.8%) in the absence of public support. In the current study, respondents' willingness to use the EMR system was comparable with previous studies [11, 16, 27]. The probable explanation for this similarity in the high proportions of people who were willing to use the EMR system might be due to the global contextual technological advancement (automation of medical systems for advancement in healthcare practice [28, 29], an increase in computer literacy, and an increase in supporting infrastructure), and the government takes high priority provided that the EMR system was included in the strategic plan of the Ministry of Health. Therefore, the result had a significant insight and implication to sustain and establish EMR system utilization in the study area as far as managers found in the study setting can work cautiously by capacitating the staff with appropriate skill and availing necessary inputs. However, the result of this study is lower than that of a study conducted in Nigeria (Lagos) [30]. The probable reason for this difference could be attributed to the variability of the study subjects being included in the studies. Health professionals included in the current study were considered from all levels of facilities while respondents in Nigeria were taken only from tertiary levels of hospital settings. Hence, the level of infrastructure, resource allocation, managerial support, and skilled manpower also varied across the health tier system that makes a difference in utilizing the EMR system.

On the other way round, the current findings were higher than those of another study conducted in Nigeria Semiurban Tertiary Hospital [31]. The possible variation existing between these two studies would be exhibited to the age difference among respondents. In the recent study, nearly half of the study subjects were found in the age group below 30 years with a mean age of 30.9 years (Table 1), whereas in the previous study, nearly three-fourths of them were in the age group above 30 years with a mean age of 35.2 years [31]. This implies the fact that the more people are younger, the more they naturally tend to have motive, interest, and commitment to accept new technology developments [19, 25, 30].

Studies found that deficit of basic and refreshment training on computers and eHealth among health professionals is the possible hindrance factor for the utilization of EMR systems in healthcare facilities that might lead to undesirable patient health outcomes [19]. This is actually reported in the same fission by the current study in that health professionals who had ever trained for the EMR system were more likely to be willing to use the EMR system than their counterparts, as supported by previous studies [10, 17, 20, 24, 25, 32]. This is also actually in line with the existing fact that training can change the knowledge, attitude, and skills of health professionals on computer systems and as a result increase commitment to use the EMR system. Thus, before the actual launching of such kind of program, it is mandatory to assess the existing level of knowledge, acceptance, and utilization habits of those subjects being studied.

Successful implementation and sustainability of an EMR system utilization in healthcare industries depend on the computer skills of all healthcare professionals who were exposed in using it [28]. Additionally, computer skill or literacy is the pillar of information communication and EMR utilization in the healthcare system; for this reason, more than half (58.3%) of the respondents in the current study were with sufficient skill of computer application, and respondents whose computer skill was sufficient were more likely willing to use the EMR system than their counterparts. This finding was in line with the study findings from Ethiopia, Adama Hospital [24] and North Gondar zone [19], Nigeria [30], Kenya [33], and Egypt [34]. This is due to the fact that those health professionals with sufficient computer skills and application could be confident enough in using EMR which indirectly influenced their views to use the EMR system. Availability of adequate computers, other resources, training center and support from non-governmental organization were also the likely explanations for the similarity of being sufficient skill.

Several studies have indicated that there is a positive relationship between the level of knowledge on EMR and willingness to use electronic medical records, and the present study, as a result, identified that health professionals who had good knowledge on EMR system were more likely willing to use the EMR system as compared to those with poor knowledge. This might be due to the fact that health professionals with good knowledge do have the tendency to accept the advantage of technology and are more likely able to willingly use the EMR system. This is supported by the study in the North Gondor zone [19], the Harer region [32], and Iran [35] and the study conducted in East Yangon General Hospital [36] where a significant relationship between the importance of EMR and use of it was pronounced. Thus, interested groups and program owners of the EMR system should strengthen continual capacity building among less knowledgeable experts in order to narrow the awareness gaps found in the health system.

Reliable and timely health information is the foundation of health systems action where information and communication technology initiatives such as EMRs enhance the decision-making process. However, it is sometimes not available when required because of poor managerial priority, budget allocation, and support. This was truly explained by the current study by the fact that managerial support and access to EMR guidelines were an independent determinant for willingness to use the EMR system. This result is similar to that of the study conducted in Ethiopia, Ayder Referral Hospital [25], and Saudi Arabia Hospital [17]. This might be explained by the fact that when managerial support is in place, more resources including working manuals would be allocated to the EMR implementation provided that professionals are motivated and eager to use the EMR package. Additionally, managerial actions would increase supportive supervision, and therefore, more staff accepted to join in utilizing the EMR system. This study could not be realized without any limitations. Among them, recall bias was the commonest one and leads to a poor estimate of results. Moreover, social desirability bias was not minimized. The study assessment relied on self-report and thus did not provide an objective measure of the healthcare experts' skill in using the EMR system.

## 5. Conclusion

Majority of the healthcare professionals showed a better willingness to use the EMR system in parallel to existing literature and national plan. Since health professionals were not the main actors of the EMR system adoption and utilization, decision personnel should take the lion's share in order to automate the e-health system in particular in the study area and in general in Ethiopia healthcare facilities. EMR program, which was recommended for expansion by the Ministry of Health nationwide, should involve and prioritize those who are computer illiterate and less knowledgeable and those unable to access the EMR guideline and managerially unsupported professionals. Enhancing and contextualizing EMR training in the healthcare curricula among universities in Ethiopia are also a beneficial step to scale up EMR system use. In addition to this, awareness creation, cultivating skills, expanding infrastructures, allocating enough resources, and changing the eye view of policy-makers towards e-health are the milestone interventions in improving the landscape of Ethiopia's health ICT. A further assessment of the effective means of increasing EMR system use in the study area is a research agenda in countries with limited resources. Future studies should be emphasized on mixed approaches of both quantitative and qualitative methods so as to have an in-depth investigation based on qualitative methods.

## Figures and Tables

**Table 1:** Sociodemographic characteristics of healthcare professionals in healthcare facilities of Bahir Dar city, Northwest Ethiopia, 2019 (*N* = 616).

Variables	Category	Frequency (*f*)	Percent (%)
Sex	Female	351	57.0
	Male	265	43.0
Age in years	20-30	293	47.6
	30-40	278	45.1
	>40	45	7.3
Religion	Orthodox	523	84.9
	Muslim	73	11.9
	Others	20	3.2
Marital status	Married	437	70.9
	Single	171	27.8
	Divorced (widow)	8	1.3
Educational level	Diploma	92	14.9
	Degree	453	73.5
	Masters and above	71	11.6
Profession	Nurse	328	53.2
	Physician	88	14.3
	Laboratory personnel	66	10.7
	Pharmacy personnel	50	8.1
	Midwives	35	5.7
	Others^∗^	49	8.0
Monthly income in birr	1561-3200	17	2.8
	3201-5250	139	22.6
	5251-7800	278	45.1
	7801-10900	165	26.8
	≥10901	17	2.8
Working experience in years	≤7	375	60.9
	>7	241	39.1

^∗^Optometrists, public health officers, anesthesiologists, and radiologists.

**Table 2:** Technical factors of health professionals in willingness to use the EMR system in facilities of Bahir Dar city, Northwest Ethiopia, 2019 (*N* = 616).

Variables	Category	Frequency (*f*)	Percent (%)
Knowledge on EMR system	Good	283	45.9
	Poor	333	54.1
Computer skill	Sufficient	359	58.3
	Not sufficient	257	41.7
EMR training	Yes	278	45.1
	No	338	54.9
Reason for not taking EMR system training	Lack of time to take training	26	7.7
	Lack of access to take training	248	73.4
	Not interested to take training	31	9.2
	My work does not need training	33	9.8
Using computer for EMR	Yes	402	65.3
	No	214	34.7
Reasons for using computer and EMR system	Report writing	46	11.4
	Seeing videos and listening to music	20	4.9
	Reading	116	28.9
	Keeping patient file and profiles	220	54.7
Language barrier	Yes	190	30.8
	No	426	69.2

**Table 3:** Organization- and resource-related factors of respondents in healthcare facilities of Bahir Dar city, Northwest, Ethiopia, 2019 (*N* = 616).

Variables	Category	Frequency (*f*)	Percent (%)
Computer access	Yes	484	78.6
	No	132	21.4
Presence of trained IT technical personnel	Yes	410	66.5
	No	206	33.5
Internet access for EMR system use	Yes	399	64.8
	No	217	35.2
EMR guideline access	Yes	538	87.3
	No	78	12.7
Adequate budget allocation	Yes	239	38.8
	No	316	51.3
	I do not know	61	9.9
Management support for EMR system	Yes	398	64.6
	No	218	35.4
M&E on use of EMR system	Yes	386	62.6
	No	230	37.4

**Table 4:** Willingness of healthcare professionals to use the EMR system in healthcare facilities of Bahir Dar city, Northwest Ethiopia, 2019 (*N* = 616).

Variables	Category	Frequency (*f*)	Percent (%)
Willingness to avail a personal computer to use for EMR system	Yes	439	71.3
	No	177	28.7
Willingness to undergo computer training to enable EMR system usage	Yes	516	83.8
	No	100	16.2
Willingness to implement EMR system after taking EMR training	Yes	451	73.2
	No	165	26.8
Willingness to use EMR system for patient service and if properly trained	Yes	451	73.2
	No	165	26.8
Willingness to use EMR system if full infrastructure is being available	Yes	492	79.9
	No	124	20.1
Willingness to use EMR system, overall	Yes	529	85.9
	No	87	14.1

**Table 5:** Factors associated with willingness to use the EMR system among healthcare professionals in healthcare facilities of Bahir Dar city, Northwest Ethiopia, 2019 (*N* = 616).

Variables	Willingness to use		OR (95%)		*P* value
	Yes	No	COR	AOR	
Got EMR training					
Yes	267	11	7.04 (3.65-13.55)	3.75 (1.73-8.12)	0.001^∗∗^
No	262	76	1	1	
EMR guideline access					
Yes	482	56	5.67 (3.34-9.66)	2.76 (1.36-5.60)	0.005^∗∗^
No	47	31	1	1	
Management support to use EMR					
Yes	369	29	4.61 (2.85-7.48)	2.59 (1.40-4.77)	0.002^∗∗^
No	160	58	1	1	
Computer skill					
Sufficient	338	21	5.56 (3.30-9.38)	2.46 (1.31-4.61)	0.005^∗∗^
Not sufficient	191	66	1	1	
Knowledge on EMR system					
Good	266	17	4.17 (2.39-7.39)	2.11 (1.02-4.37)	0.044^∗^
Poor	263	70	1	1	

^∗^Statistically significant at *P* < 0.05. ^∗∗^Significant at *P* < 0.01.

## Data Availability

The datasets analyzed during the current study are available from the corresponding author on reasonable request.

## Abbreviations

EMR: Electromedical records
SPSS: Statistical Package for Social Science
AOR: Adjusted odds ratio
CI: Confidence interval
COR: Crude odds ratio
ICT: Information and communication technology
WHO: World Health Organization
US: United States
M&E: Monitoring and evaluation.

## References

[1] Walsham G. (2020). Health information systems in developing countries: some reflections on information for action. *Information Technology for Development*.

[2] Evans R. S. (2018). Electronic health records: then, now, and in the future. *Yearbook of Medical Informatics*.

[3] World Health Organization (2012). *Management of patient information trends and challenges in member states*.

[4] Lynn W., Joy M., Rogers S. (2017). Impact of electronic medical records on healthcare delivery in Kisii Teaching and Referral Hospital. *Medical and Clinical Review*.

[5] Bazile E. P. (2016). *Electronic Medical Records (EMR): An Empirical Testing of Factors Contributing to Healthcare Professionals’ Resistance to Use EMR Systems: A Doctoral Desertation*.

[6] Tsai J., Bond G. (2007). A comparison of electronic records to paper records in mental health centers. *International Journal for Quality in Health Care*.

[7] Lee J., Kuo Y. F., Goodwin J. S. (2013). The effect of electronic medical record adoption on outcomes in US hospitals. *BMC Health Services Research*.

[8] Fairley C. K., Vodstrcil L. A., Huffam S. (2013). Evaluation of electronic medical record (EMR) at large urban primary care sexual health centre. *PLoS One*.

[9] Stone C. P. (2014). *Glimpse at EHR Implementation Around the World: The Lessons the US Can Learn*.

[10] Mkalira Msiska K. E., Kumitawa A., Kumwenda B. (2017). Factors affecting the utilisation of electronic medical records system in Malawian central hospitals. *Malawi Medical Journal*.

[11] Almulhem J. A. (2017). *Layperson perceptions and attitudes towards a national electronic health record introduction in Saudi Arabia: theses and dissertations*.

[12] Garcia-Smith D., Effken J. A. (2013). Development and initial evaluation of the clinical information systems success model (CISSM). *International Journal of Medical Informatics*.

[13] Boonstra A. B., Broekhuis M. (2010). Barriers to the acceptance of electronic medical records by physicians from systematic review to taxonomy and interventions. *BMC Health Services Research*.

[14] Msukwa M. K. B. (2011). *User perceptions on electronic medical record system (EMR) in Malawi: a doctoraldesertation*.

[15] Terry A. L., Thorpe C. F., Giles G. (2008). Implementing electronic health records key factors in primary care. *Canadian Family Physician*.

[16] Uwambaye P., Njunwa K., Nuhu A. (2017). Health care consumer’s perception of the Electronic Medical Record (EMR) system within a referral hospital in Kigali, Rwanda. *Rwanda Journal*.

[17] Aldosari B., Al-Mansour S., Aldosari H., Alanazi A. (2018). Assessment of factors influencing nurses acceptance of electronic medical record in a Saudi Arabia hospital. *Informatics in Medicine Unlocked*.

[18] Abdulmalek L. J. Doctors knowledge, attitude and practice towards electronic medical records in the general hospitals in Benghazi.

[19] Biruk S., Yilma T., Andualem M., Tilahun B. (2014). Health Professionals' readiness to implement electronic medical record system at three hospitals in Ethiopia: a cross sectional study. *BMC Medical Informatics and Decision Making*.

[20] Tilahun B., Fritz F. (2015). Comprehensive evaluation of electronic medical record system use and user satisfaction at five low-resource setting hospitals in Ethiopia. *JMIR Medical Informatics*.

[21] Qureshi Q. A., Shah B., Khan N., Miankhel K., Nawaz A. (2012). Determining the users’ willingness to adopt electronic health records (EHR) in developing countries. *Gomal University Journal of Research*.

[22] Sukums F., Mensah N., Mpembeni R., Kaltschmidt J., Haefeli W. E., Blank A. (2014). Health workers’ knowledge of and attitudes towards computer applications in rural African health facilities. *Glob Health Action*.

[23] Saleh S., Khodor R., Alameddine M., Baroud M. (2016). Readiness of healthcare providers for eHealth: the case from primary healthcare centers in Lebanon. *BMC health Services Research*.

[24] Alemayehu D. (2015). Investigating electro medical record (smart cares oftware) implementation in a healthcare facility. *A a guiding framework for Adama hospital Hospital medical Medical College, [M.S. thesis]*.

[25] Yehualashet G., Asemahagn M., Tilahun B. (2015). The attitude towards and use of electronic medical record system by health professionals at a referral hospital in Northern Ethiopia: cross-sectional study. *Journal of Health Informatics in Africa*.

[26] Khoja S., Scott R., Ishaq A., Mohsin M. (2017). Testing reliability of eHealth readiness assessment tools for developing countries. *E-health International Journal*.

[27] Hasanain R. A. (2015). *Development of an EMR implementation framework for public hospitals in Saudi Arabia, [Ph.D. thesis]*.

[28] Alkathiri N. M. (2016). *Ubiquitous electronic medical record (EMR) for developing countries, [M.S. thesis]*.

[29] Ajami S., Ketabi S., Isfahani S., Heidari A. (2011). Readiness assessment of electronic health records implementation. *Acta Informatica Medica*.

[30] Onigbogi O., Poluyi A. O., Poluyi C. O., Onigbogi M. O. (2018). Doctors’ attitude and willingness to use electronic medical records at the Lagos University Teaching Hospital, Lagos, Nigeria. *Online Journal of Public Health Informatics*.

[31] Adebara O. V., Adebara I. O., Olaide R., Emmanuel G. O., Olanrewaju O. (2017). Knowledge, attitude and willingness to use mHealth technology among doctors at a semi urban tertiary hospital in Nigeria. *Journal of Advances in Medicine and Medical Research*.

[32] Alwan K., Awoke T., Tilahun B. (2015). Knowledge and utilization of computers among health professionals in a developing country: a cross-sectional study. *JMIR Human Factors*.

[33] Peter N., Wanja M., Susan N. (2018). Technical factors affecting electronic medical record system information use: a case of Kakamega County Referral Hospital out patient department. *IOSR Journal of Nursing and Health Science (IOSR-JNHS)*.

[34] Eldin A. S., Saad D., Samie G. A. (2013). Evaluation of electronic health records adoption in Egypt. *International Journal of Engineering Research and Applications*.

[35] Garavand A., Samadbeik M., Asadi H., Abhari S. (2016). Readiness of Shiraz teaching hospitals to implement Electronic Medical Record (EMR). *Journal of Health Management & Informatics*.

[36] Htoo P. S. (2017). *Health professionals’ readiness to implement electronic medical record system at East Yangon general hospital, [M.S. Thesis]*.

